# Epitheliotropic Infections in Wildlife Ruminants From the Central Alps and Stelvio National Park

**DOI:** 10.3389/fvets.2020.00229

**Published:** 2020-04-30

**Authors:** Laura Gallina, Federica Savini, Giovanni Casà, Irene Bertoletti, Alessandro Bianchi, Lucia Rita Gibelli, Davide Lelli, Antonio Lavazza, Alessandra Scagliarini

**Affiliations:** ^1^Dipartimento di Scienze Mediche Veterinarie, Alma Mater Studiorum, Università di Bologna, Bologna, Italy; ^2^Istituto Zooprofilattico Sperimentale della Lombardia e dell'Emilia-Romagna, Brescia, Italy; ^3^Dipartimento di Medicina Specialistica Diagnostica e Sperimentale, Alma Mater Studiorum, Università di Bologna, Bologna, Italy

**Keywords:** coinfection, papillomavirus, poxvirus, ruminants, wildlife, epitheliotropic, phylogenesis

## Abstract

The mountain chain of the Alps, represents the habitat of alpine fauna where the red deer (*Cervus elaphus*) population is the outmost numerous, followed by the chamois (*Rupicapra rupicapra*) and the alpine ibex (*Capra ibex*) at higher altitudes. Previous reports showed the circulation of epitheliotropic viruses, belonging to the families *Papillomaviridae* and *Poxviridae*, causing skin and mucosal lesions in wild ruminants of the Stelvio National Park, situated in the area. To deepen our knowledge on the natural dynamics of the infections, a passive surveillance on all the cases of proliferative skin and mucosal lesions in wild ruminants was performed. Twenty-seven samples (11 chamois, 10 red deer and 6 ibex) collected from 2008 to 2018 were analyzed by negative staining electron microscopy, histology, and PCR followed by genome sequencing and phylogenetic analyses. Results confirmed the spread of Parapoxvirus of Red Deer in New Zealand (PVNZ) in Italy, and its ability to cause severe lesions i.e., erosions and ulcers in the mouth. We showed for the first time a PVNZ/CePV1v (C*. elaphus* papillomavirus 1 variant) co-infection identified in one red deer. This result supports previous evidence on the ability of papillomavirus and parapoxvirus to mutually infect the same host tissue. Interestingly two ibex and one chamois showing orf virus (OV) skin lesions were shown to be co-infected with bovine papillomavirus type 1 and 2. The presence of bovine papillomavirus, in orf virus induced lesions of chamois and ibex raises the question of its pathogenetic role in these animal species. For the first time, OV/CePV1v co-infection was demonstrated in another chamois. CePV1v is sporadically reported in red deer throughout Europe and is considered species specific, its identification in a chamois suggests its ability of cross-infecting different animal species. Poxviruses and papillomavirus have been simultaneously detected also in the skin lesions of cattle, bird and human suggesting a possible advantageous interaction between these viruses. Taken together, our findings add further information on the epidemiology and pathogenetic role of epitheliotropic viruses in wild ruminants living in the central Alps and in Stelvio National Park.

## Introduction

Epitheliotropic viruses belonging to the *Poxviridae* and *Papillomaviridae* families include a number of viral species which are known to infect wild ruminants in many parts of the world. According to ICTV in the *Poxviridae* family, *Chordopoxvirinae* subfamily, the genera Deerpoxvirus and Parapoxvirus include several species able to cause diseases in wild ruminants (1). The viruses belonging to the genus Deerpoxvirus (DPV) are responsible for non-parapoxvirus-like infections in the members of two subfamilies of cervids, American deer (*Odocoileinae*) and reindeer (*Rangiferinae*). These viruses, resembling orthopoxviruses in shape, have been reported in North America in wild mule deer (*Odocoileus hemionus*) in Wyoming (2), black-tailed deer (*O. h. columbianus*) (3) and pudu (*Pudu puda*) (4) in California, in wild cervid species in the Northwest Pacific (5), in farmed white-tailed deer in Mississipi (6) and in a goitered gazelle (*Gazella subgutturosa*) (7). The first genome sequence of Mule Deerpoxvirus (MDPV), from a farmed white-tailed deer fawn in Florida, has been recently published by Sayler et al. (8). A sero-survey, performed in Oregon, indicated that the exposure to DPV is common in Odocoileus populations leading to speculate that the high antibody prevalence and the low rate of observed DPV-related disease may suggest a low virulence of these pathogens (9).

To date, in the genus Parapoxvirus (PPV) there are four confirmed PPV species recognized by the ICTV: the prototype member orf virus (ORFV) endemic in most sheep- and goat-raising countries, the bovine popular stomatitis virus (BPSV) and pseudocowpox virus (PCPV) mainly infecting cattle. The Parapoxvirus of Red Deer in New Zealand (PVNZ), described by Robinson and Mercer (10) has now become the fourth PPV species (11). OV, PCPV, and PVNZ have been detected in several deer species including: white-tailed deer from the United States (12, 13), red deer (*Cervus elaphus*) in New Zealand, Italy and Germany (10, 11, 14, 15), chamois (*Rupicapra rupicapra*) in Italy and Austria (16, 53) and ibex (*Capra ibex*) in Italy (14) and reindeer (*Rangifer tarandus*) in Scandinavia (17, 18). In 2018, Tryland and collaborators reported parapoxvirus infections in caribou and Sitka black-tailed deer confirming that most of the wild ruminant species in Alaska can be affected by OV. Human infection from cervid-borne parapoxviruses (OV and PCPV) has been reported in America and in Europe (12, 16, 19, 20). These findings showed that hunters, wildlife inspectors, subsistence harvesters, and biologists are the professional categories most exposed to PPV infections from wild animals.

The family *Papillomaviridae* comprises 330 PV types currently listed as Reference Genomes for animals in the Papillomavirus Episteme (http://pave.niaid.nih.gov). Only 39 ruminant PV types are recognized up to now [Papillomavirus Episteme, (21)]. Within the large group of ungulates (diverse group of mammals that includes odd-toed and even-toed ungulates), bovine papillomavirus (BPV) plays a major role in a variety of diseases in domestic and wild ruminants (22). PV DNAs have been detected in Europe and North America in papilloma lesions of several wild deer species (23–31). The majority of papillomaviruses detected in wild deer belong to the Delta genus and cause fibropapillomatosis. This disease has been reported in roe deer (*Capreolus capreolus*) in Hungary and the Czech Republic (28, 29) and in red deer in Italy and Portugal (32, 33). The causative agents CcaPV1 and CePV1v are closely related PV variants of the Delta papillomavirus genus. At present, in all the European countries where red deer fibropapillomas have been reported, there was no evidence of a similar infection in roe deer. To date, Hungary is the only European country in which there is a coexistence of CePV1v and CcaPV1 infection in local deer population (32). A new papillomavirus, RtPV2, genus Xi, was identified in the eye swab samples of semi-domesticated reindeer (*R. tarandus tarandus)* in Norway (34). More recently, multiple persistent pigmented squamous papillomas (warts) on the chins in European red (*C. elaphus elaphus*) X wapiti (*C. e. canadensis*) deer stags were shown to be caused by CePV1 belonging to the Epsilon genus (35).

Wildlife disease surveillance is a complementary component of human and animal disease surveillance, monitoring, prevention and control programs, as well as conservation efforts (36). Smits et al. (34) underlined the importance of studying the virus diversity in wildlife to provide important epidemiological baseline information about pathogens and their evolutionary patterns in different host species. A collaboration network of hunters, wildlife rangers, Istituto Zooprofilattico Sperimentale della Lombardia e dell'Emilia Romagna (IZSLER) and University of Bologna was established to better understand the natural dynamics and possibility for interspecies transmission of the infections and clinical diseases caused by the epitheliotropic viruses circulating in wildlife population of the central Alps and Stelvio Park. With this purposes, a range of testing were employed for confirmatory diagnosis and the main findings on viral genomic characterization are presented.

## Materials and Methods

### Sampling

The study was performed in northern Italy, Lombardia region in the Valtellina valley (province of Sondrio), Brembana and Seriana valley (province of Bergamo) and in Camonica valley (province of Brescia) where wild ruminants such as chamois (*R. rupicapra*), red deer (*C. elaphus*) and ibex (*C. ibex*) are present. samples were taken from carcasses of necropsied animals, during the diagnostic activities of the IZSLER. In fact, chamois and ibex found dead are commonly submitted to IZSLER and examined in the framework of the regional health monitoring and control plan for wildlife (DDG, 5 December 2012, no. 11358).

A certain number of red deer were randomly culled during a program aimed at controlling its population density in the Stelvio National Park (Lombardia Region).

Moreover, selective hunting of wild ungulates is legal during the September-December period. In accordance with Italian Law (N. 157 of 11/02/1992), hunters must carry culled wild ruminants to the control centers where age, sex and morpho-biometric measurements are registered and gross lesions inspection of carcass and organs are also performed.

For this study, cutaneous pathological samples with proliferative lesions such as epithelial or fibroepithelial neoplasms were collected from animals of every age group and adult animals during post-mortem inspection and stored at −20°C for 1–2 weeks in the control center facility and subsequently transferred at −80°C to the laboratory until further processing. Cutaneous samples (*n* = 27) were collected during seven (2008, 2010–2013, 2016, 2018) hunting seasons (Table 1). In particular, during the same period a total of 1,572 carcasses were examined 1,119 of which were red deer, 400 were chamois and 53 were ibex. The number of cases presenting skin and mucosal lesions were 19 for red deer, 20 for chamois and 9 for ibex. Samples were obtained only from legally hunted animals or animals found dead. No animal was deliberately culled for this study.

**Table 1:** List of examined cases.

**Animal ID**	**Animal species**	**EM**	**PPV (GenBank accession n.)**	**PV (GenBank accession n.)**	**Site of collection**	**Date of collection**
1126B	Red deer (F, 6 m)	PPV	MN977289 MN977284	–	Bormio (So)	08/11/2010 dead
1126C	Red deer (M, 6 m)	–	MN977290 MN977285	MN977312 MN977315	Bormio (So)	08/11/2010 dead
1126D	Red deer (F, 10 Y)	PPV	MN977293	–	Bormio (So)	08/11/2010 dead
1126E	Red deer (M, 10 m)	–	MN977291	–	Bormio (So)	08/11/2010 dead
523	Red deer (M, U)	PPV	MN977292 MN977286	–	Ponte di Legno (Bs)	23/04/2013 U
376	Red deer (M, 2 Y)	PV	–	MN985322	Vione (Bs)	03/11/2012 dead
377	Red deer (U, U)	PV	–	MN985323	Lenno (Co)	29/10/2012 hunted
1601	Red deer (U, U)	PV	–	MN977311 MN977318	Monno (Bs)	23/09/2018 hunted
1635	Red deer (M, 1 Y)	PV	–	MN977310 MN977317	Gravedona (Co)	02/09/2016 hunted
1636	Red deer (M, 1 Y)	PV	–	MN977316 MN977309	Valdidentro (So)	03/09/2016 hunted
45	Chamois (M, 2 Y)	PPV	MN977219 MN977305 MN977270 MN977209	MN977319	Sondalo (So)	03/03/2018 dead
115	Chamois (F, 1 Y)	PPV	MN977216 MN977299 MN977279 MN977210	–	Carona (Bg)	19/10/2011 hunted
265	Chamois (U, 6 m)	PPV	MN977217 MN977278 MN977200	–	Lenna (Bg)	29/11/2011 U
375	Chamois (M, 1,5 Y)	PPV	MN977221 MN977302 MN977272 MN977201	MN977313 MN977314	Val Masino (So)	08/10/2012 dead
519	Chamois (F, 6 m)	PPV	MN977222 MN977295 MN977273 MN977202	–	Ponte in Valtellina (So)	09/01/2013 dead
520	Chamois (F, 1 Y)	PPV	MN977223 MN977303 MN977274 MN977203	–	Ponte di Legno (Bs)	22/01/2013 dead
521	Chamois (F, 1 Y)	PPV	MN977224 MN977307 MN977275 MN977204	–	Ponte di Legno (Bs)	23/02/2013 dead
522	Chamois (M, 6 m)	PPV	MN977225 MN977297 MN977276 MN977205	MN977322	Val Masino (So)	27/02/2013 dead
1637	Chamois (M, 2 Y)	PPV	MN977226 MN977308 MN977287 MN977208	–	Valdidentro (So)	22/10/2018 hunted
257/09	Chamois (M, 1 Y)	PPV	HQ239071^*^ MN977294 MN977283 MN977196	–	Valdidentro (So)	19/02/2008 hunted
485/09	Chamois (F, 6 m)	PPV	HQ239073^*^ MN977298 MN977280 MN977197	–	Fusine (So)	06/03/2008 dead
07/11	Ibex (M, 8 m)	PPV	MN977213 MN977277 MN977198	–	Valbondione (Bg)	19/01/2011 dead
116	Ibex (M, 3 m)	PPV	MN977214 MN977300 MN977288 MN977211	–	Gerola Alta (So)	21/08/2011 dead
264	Ibex (U, U)	PPV	MN977215 MN977301 MN977282 MN977199	–	Valbondione (Bg)	24/10/2011 dead
44	Ibex (M, 13 Y)	PPV	MN977218 MN977304 MN977269 MN977206	MN977321	Livigno (So)	03/03/2018 dead
373/08	Ibex (M, 6 m)	PPV	HQ239072^*^ MN977296 MN977281 MN977212	–	Ponte di Lgno (Bs)	19/02/2008 dead
47	Ibex (F, 3 Y)	–	MN977220 MN977306 MN977271 MN977207	MN977320	Valfurva (So)	05/06/2018 dead

Electron microscopy (EM) results, GenBank accession numbers of the sequenced samples, site of collection and date and cause (hunted or found dead) of death of the animals. (−): negative result. (^*^): sequence already published in Scagliarini et al. (14). F, Female; M, Male; m, months; Y, years old; U, unknown.

### Histopathology

Tissue samples were fixed by immersion in 10% neutral buffered formalin and embedded in paraffin wax. Sections were cut at 4 μm, stained with hematoxylin and eosin (HE) and examined by light microscopy.

### Negative Staining Electron Microscopy

Tissue samples were subjected to negative staining electron microscopy using the Airfuge method (37). Samples were ultracentrifuged (Airfuge, Beckman Coulter Inc. Life Sciences, Indianapolis, Indiana, USA) for 15 min at 82,000 × g using a rotor holding six 175-μl test tubes in which specific adapters for 3 mm carbon-coated Formvar copper grids were placed. The grids were then stained using 2% sodium phosphotungstate (pH 6.8) for 1.5 min and observed under a Tecnai G2 Spirit Biotwin transmission electron microscope (FEI, Hillsboro, Oregon, USA) at 20,500–43,000 × for at least 15 min before being considered negative. Attempts to identify the observed viral particles were based on their morphological characteristics.

### PCR and Sequencing

DNA from 25 mg of each fresh sample was extracted using NucleoSpin Tissue kit (Macherey-Nagel GmbH, Duren, Germany). The PCR reactions were performed with a Taq PCR Master Mix Kit (Qiagen, Hilden, Germany) on a final volume of 25 ul containing 1x PCR buffer, 5 μL of Q-solution, 0.8 uM deoxynucleotide mix (Thermo Scientific, Waltham, Massachusetts, USA), 0.4 uM of sense and antisense primer, 2.5 μL of DNA template, and 0.65 units of Taq DNA polymerase. Each reaction included a no-template control and an appropriate positive control (a previously sequenced sample). The presence of BPV1 and BPV2 was tested by amplification of the L1 ORF encoding the L1 major capsid protein. The entire genome of CePV1v was obtained from two red deer samples (376 and 377) as already described (32) while for the samples 1,601, 1,635, and 1,636 only the L1 and partial L2 samples were amplified and sequenced. Detection of parapoxvirus was performed amplifying the B2L gene encoding the homolog of the vaccinia virus major envelope antigen p37K. Further genomic characterization of the Parapoxvirus positive samples was accomplished through amplification of three virulence genes GIF, vIL-10, vVEGF. Primers used and target genes amplified are listed in Table 2. Sanger sequencing was performed on both strands of the amplified DNA (ABI Prism 3100 Genetic Analyzer, Perkin-Elmer Applied Biosystems, Foster City, California, USA) and chromatograms were assembled using 4Peaks 1.7.1 (Nucleobytes 2015). Sequences were deposited in GenBank using the National Center for Biotechnology Information (NCBI) BankIt v3.0 submission tool (NCBI 2015) under accessions (see Table 1).

**Table 2:** Primers targeting different gene regions were used to detect the papillomavirus CePV1v, BPV1, BPV2, and the parapoxvirus OV and PVNZ from 27 skin lesions' samples.

**Gene**	**Acronym**	**Primers**	**Product (bp)**	**References**
Putative viral envelope antigen (OV)	B2L	PPP-1 5′-GTCGTCCACGAGCAGCT-3′ PPP-4 5′-TACGTGGGAAGCGCCTCGCT-3′	595	(38)
Granulocyte–macrophage-colony-stimulating factor/ interleukin-2 inhibition factor (OV)	GIF	GIF 5 5′-GCTCTAGGAAAGATGGCGTG-3′ GIF 6 5′-GTACTCCTGGCTGAAGAG CG-3′	408	(39)
Viral interleukin 10 ortholog (OV)	vIL-10	vIL-10-3 5′-ATGCTACTCACACAGTCGCTCC-3′ vIL-10-4 5′-TATGTCGAACTCGCTCATGGCC-3′	300	(39)
Vascular endothelial growth factor gene of OV NZ-2 like (OV)	VEGF-E	VEGF_forNZ2 5′ATGARGTTGCTCGTCKGCATAC-3′ VEGF_rev1NZ2 5′-CGTCTTCTGGGCGGCCTTGT-3′ VEGF_rev2NZ2 5′-CTTCGGCGCCGTCTAGGC-3′	399	This study
Vascular endothelial growth factor gene of (OV)	VEGF-E	GF1 5′-GCGGGATCCGCCATGAAGTTGCTCGT-3′ GF2 5′-GCGGAATTCCTAGCGGCGTCTTCTGG-3′	399	(40)
Vascular endothelial growth factor gene of (PVNZ)	VEGF-E	5′-TTTGGCGCGCCAGAGACTTCTAATACAGTGTAGCG-3′ 5′-TCACCCGAACGCGTACGTCTTGGAGGCATAG-3′	447	(41)
Vascular endothelial growth factor NZ7 like gene (OV)	VEGF-E	GF3 5′-GCGGGATCCACGATGAAGTTAACAGC-3′ GF4 5′-GCGGAATTCTTATCGTCTAGGTTCCCTA-3′	450	(40)
minor capsid protein (CePV1v)	L2	CePVL2F 5′-TAGACTACTACTACCTGTGACACAC-3′ CePVL2R 5′-TGGTCACAGGTGTAGGTGGCA-3′	250	(33)
major capsid protein (CePV1v)	L1	CePVL1F5731 5′-TATTTGCCACCTACACCTGTGAC-3′ CePVR7253 5′-CAGCTGGACAGCTCATTAG-3′	1522	(33)
E5 oncoprotein (BPV1 and BPV2)	E5	5′B1/2-E5: 5′-CACTACCTCCTGGAATGAACATTTCC-3′ 3′B1/2-E5: 5′-CTACCTTWGGTATCACATCTGGTGG-3′	499	(42)
major capsid protein (BPV1)	L1	BPV1estL1f 5′-TGATGGGCACACAGTTGATTTGTAC-3′ BPV1estL1r 5′-GGTGCAGTTGACTTACCTTCTGT-3′	1621	This study

### Phylogenetic Analysis

Phylogenetic analyses were performed by MEGA 7. The evolutionary history for the B2L and vVEGF genes and concatenated B2L, GIF, vIL-10, and vVEGF genes were inferred by using the maximum Likelihood method based on Tamura 3-parameter model and gamma-distribution. Statistical support for branches of the trees was evaluated by bootstrapping with 1,000 replications. The concatenated phylogenetic tree has been performed based on the alignment of 19 amino acid sequences, there were a total of 569 positions in the final dataset. The analysis of the B2L gene involved 62 nucleotide sequences and there were a total of 489 positions in the dataset. The vVEGF phylogenetic tree has been performed based on 53 deduced amino acid sequences. There were a total of 111 positions in the final dataset.

## Results

### Gross Pathology and Histopathology

Overall, a 3% prevalence of skin and mucosal lesions attributable to PPV or PV was calculated in the years considered. The prevalence for each animal species is different, ibexes showed the highest prevalence (17%) followed by chamois (5%) and deer (1.7%). The lesions are distributed differently depending on the age groups, in particular animals of the year (*N* = 8, 36%) and up to 2 years of age (*N* = 11, 50%) account for 86% of positive cases, while in adults PPV and PV lesions are less frequent (*N* = 3, 14%).

**Figure 1:**
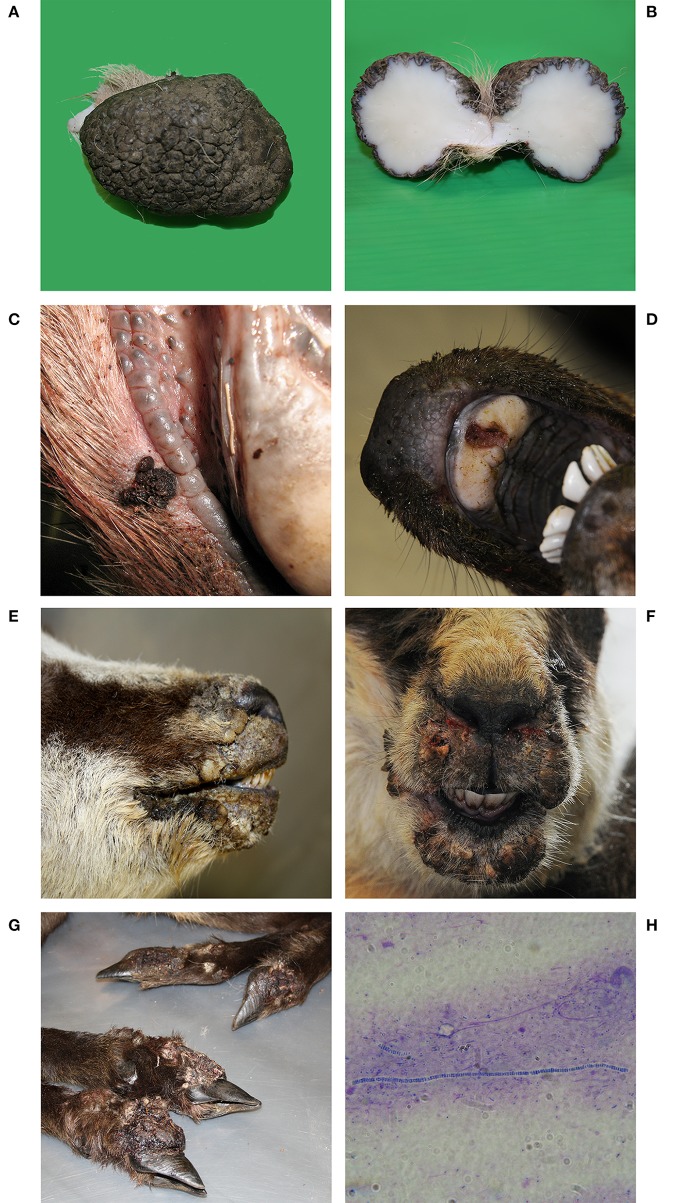
Red deer N° 1635. **(A)** Pedunculated, oval cutaneous neoformation of dimensions 7 × 5 × 5 cm. Hyperkeratotic, glabrous, black, irregular, and fissured surface. **(B)** When cut, lardaceous consistency, white color and uniform, regular appearance. **(C)** Red deer N°1126D and **(D)** Red deer N° 523. Proliferative lesions, erosions, and ulcers on the lips and on the hard palate. **(E)** Chamois N° 522 and **(F)** Chamois N° 520. Ulcerative and crusting lesions on the mucocutaneous tissues of the head: Chamois N° 375. **(G)** Severe proliferative pododermatitis of all four limbs with coronary hyperplasia. **(H)** Microscopic detection of *Dermatophilus congolensis*. Gram staining, 1000x.

Post-mortem inspection showed different size firm cutaneous tumors with rough hairless pigmented surface, resembling papillomas (Figures 1A,B), on the hind legs of 5 hunted red deer (376, 377, 1,601, 1,635, 1,636). On histological examination, exophytic proliferation with hyperparakeratosis and pseudo-epitheliomatous hyperplasia was observed. The stratum granulosum was prominent with presence of large, rounded and irregular keratohyalin granules in the epidermis and the dermis showed a proliferation of fibroblasts and connective tissue with multifocal mononuclear inflammatory infiltrate in all the papilloma-like lesions of Red deer. The histopathological findings were consistent with red deer fibropapillomatosis.

Proliferative lesions, erosions, and ulcers, resembling parapoxvirus infections, on the lips and on the hard palate (Figures 1C,D) were reported in 5 Red Deer found dead (1126B, 1126C, 1126D, 1126E, 523), in seven Chamois (45, 265, 520, 521, 1,637, 257/09, 485/09) and three ibexes (47, 7/11, 373/08) (found dead). Ulcerative and crusting lesions on the coronary band as well as the mucocutaneous tissues of the head (Figures 1E,F) have been shown in four chamois (115, 375, 519, 522) and two ibexes (116, 264). PPVs' typical hyperplastic and proliferative epidermal lesions with vacuolation and swelling of keratinocytes in the stratum spinosum and intracytoplasmic eosinophilic inclusion bodies were observed in all the affected animals. In chamois 375 proliferative dermatitis generally referred to as strawberry footrot (43) was observed in the four lower legs (Figure 1G). The bacteriological examination confirmed the isolation of *Dermatophilus congolensis* (Figure 1H).

### Negative Staining Electron Microscopy

**Figure 2:**
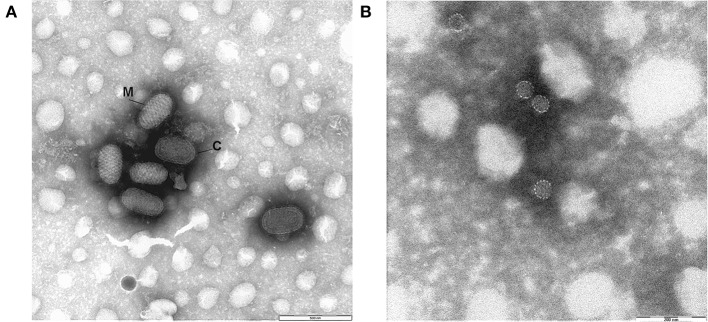
Negative-staining EM micrographs of **(A)** Parapoxvirus showing the typical morphology: oblong, rounded, or ovoid large particles of around 140–170 nm wide and 220–300 nm long. M (mulberry) form, C (capsule) form. Bar = 500 nm. **(B)** Papillomavirus: non-enveloped icosahedral particles of around 50–55 nm in diameter. Bar = 200 nm.

Negative-staining EM showed the presence of parapoxvirus in 19 samples (3 red deer, 11 chamois, 5 ibex) out of 27 cases examined (Table 1). PPV identification was based on the typical morphology: oblong, rounded, or ovoid large particles (140–170 nm wide and 220–300 nm long). Either the characteristic M (mulberry) form, showing regular surface structure where tubules with a diameter of 10–20 nm form a criss-cross pattern, and less frequently the C (capsule) form showing a smooth uniform surface and a thick membrane were observed (Figure 2A).

Indeed, with the same technique non-enveloped icosahedral particles of around 50–55 nm in diameter, morphologically referable to PVs (Figure 2B) were found in 5 red deer samples. Only in three samples, 2 red deer and 1 ibex, we did not observe any viral particles by NSEM.

### PCR and Sequencing

PCR amplification and subsequent sequencing of red deer samples revealed PVNZ and CePV1v infections in 5 and 6 samples respectively. Sequencing of a 550 bp of the B2L gene showed 100% nucleotide identity not only among the four PVNZ isolates but also with the cases previously reported in the same geographical area (14). The analysis of the PVNZ VEGF gene revealed 100% amino acidic identity between the samples1126B and 1126C and 95.9% with the sample 523. It was not possible to amplify PVNZ VEGF from the samples 1126E and 1126D. CePV-1v was detected in the form of a single infection in five skin samples (376, 377, 1,601, 1,635, 1,636) and as coinfecting agent together with PVNZ in the sample 1126C. The entirely sequenced 376 and 377 samples showed 100% nucleotide identity with the IT-1127 CePV1v (GenBank n. JQ744282.1). Sequencing of the entire L1 gene (1,524 bp) of the samples 1601, 1635, 1636, 1126C, showed nucleotide identity ranging from 99.9 to 100% among all the analyzed sequences. The amplification and sequencing of 274 bp of the CePV1v L2 gene revealed a nine-nucleotide deletion, causing the lack of three amino acids of the encoded capsid protein. All samples obtained from chamois and ibex tested positive for parapoxvirus infection. Sequencing of the 550 bp of the B2L gene identified OV as the causative agent of the lesions with nucleotide and amino acid identity ranging from 96.3 to 100% in the chamois strains and 97.2 to 100% for the ibex strains. Sequencing of the vIL-10 gene from chamoix and ibex samples allowed to obtain 410 bp sequences displaying 89.1 to 100% and 80.1 to 100% nucleotide and amino acid identity, respectively. The GIF gene was sequenced from all the ibex and chamois samples producing 350 nucleotide sequences showing 83.5 to 100% identity among all isolates. Amplification and sequencing of the entire vVEGF gene identified NZ-2-like VEGF variants in all samples but one (257) that showed high similarity to NZ7-like VEGF (GenBank n. S67522). Ibex and chamois vVEGF gene showed high rate sequence variation in particular, samples 116, 264, 265, 7/11 revealed a 21 nucleotides insertion in the N-terminal region while a 12 nucleotides insertion was evidenced in the C-terminal region of the 115, 116, 44, 1,637 samples. In two chamois (45, 522) and two ibexes (44, 45) it was possible to detect BPV1 co-infecting an OV lesion by sequencing the L1 gene. Finally, in chamois 375 showing echtyma lesion on the nasolabial rim, it was possible to identify and sequence the entire L1 sequence of CePV1v.

### Phylogenetic Analysis

**Figure 3:**
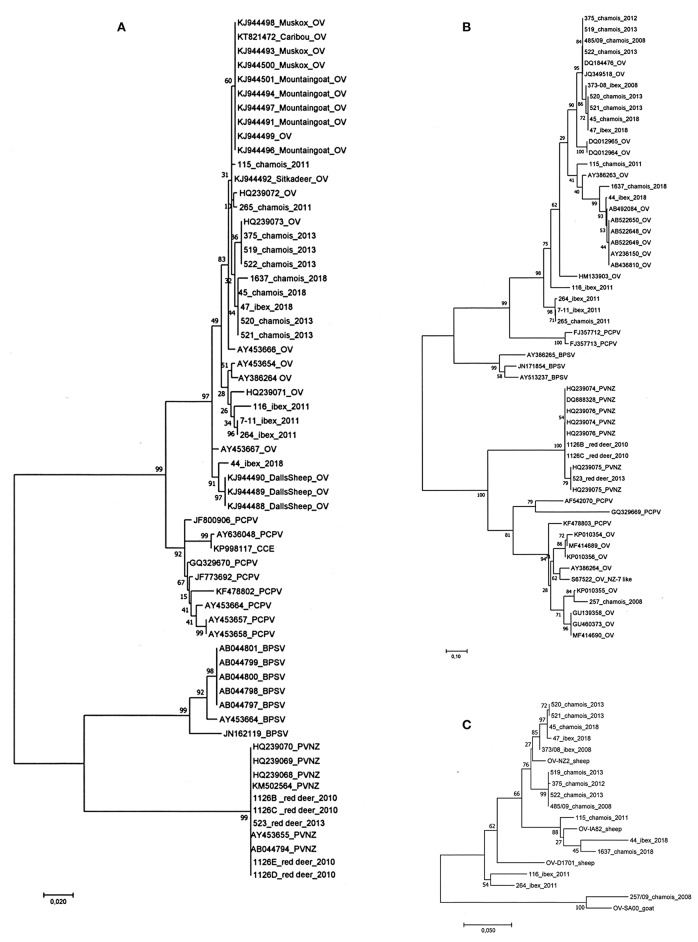
Phylogenetic analysis performed on the parapoxvirus genes sequenced. **(A)** The phylogenetic tree of the partial B2L gene of the 22 parapoxvirus samples showing clusters corresponding to the four species of the parapoxvirus genus, all the OV sequences from ibex and chamois cluster with other strains isolated from sheep and goats. Five PVNZ isolates grouped into a separate cluster with other PVNZ. **(B)** The phylogenetic analysis of the vVEGF showed an expected high variability of the vVEGF variant of isolates from chamois and ibex. The chamois sample 257 grouped with the OV NZ7 like vVEGF variants. **(C)** Phylogenetic tree based on the concatenated B2L, GIF, vVEGF, and vIL10 amino acid sequences. No host-dependent clustering was identified instead two main clusters of NZ2 and NZ7 OV variants. (OV-NZ-2: GenBank n. DQ184476 OV-IA82 GenBank n. AY386263 D1701 GenBank n. HM133903 OV-SA00 GenBank n. AY386264).

The phylogenetic analysis of the partial B2L gene of the 22 parapoxvirus samples compared to the sequences of the four species of the parapoxvirus genus, grouped them in two viral species' clusters (Figure 3A). Comparison of the 17 sequences from ibex and chamois showed that they were OV and that they cluster with other OV strains isolated from sheep and goats regardless of the infected animal species as already reported for other wildlife species such as Mountain goat (*Oreamnos americanus*), Dall's sheep (*Ovis dalli dalli*), muskoxen (*Ovibos moschatus*), caribou (*R. tarandus*). As expected the five PVNZ cases grouped into a separate cluster with three other PVNZ isolated from red deer in the Stelvio National park (14). The phylogenetic analysis of the deduced amino acid sequences obtained from the vVEGF gene showed the high variability of viral isolates from chamois and ibex confirming clustering with OVs (data are showed in Figure 3B). In particular, the sample from chamois 257 grouped with the OV NZ7 like VEGF variants (Figure 3B). In accordance with previous data, vVEGF from PVNZ demonstrated a lower degree of sequence variability compared to the other parapoxvirus species grouping in a separate cluster (Figure 3B). Despite the variability identified in the B2L, GIF, vVEGF, and vIL10 genes from OV infecting ibex and chamois, the phylogenetic analysis performed on the deduced amino acid sequences of the four OV concatenated genes did not show host-dependent clusterization and revealed two main clusters with the chamois 257 grouping with the NZ7 like strain SA00 isolated from a goat (Figure 3C).

## Discussion

Wildlife infectious diseases play a major role in regulating natural populations and may seriously threaten the health and welfare of wildlife, livestock and humans. We and others previously reported the circulation of epitheliotropic viruses in wild ruminant populations in different European countries. Several cases of proliferative skin and mucosal diseases associated to CePV1v (32, 33), PVNZ and OV (14) have been reported in wild ruminants of the Stelvio Park which is located in the central Alps. These diseases have been sporadically detected in hunted animals and carcasses of animals found dead during passive surveillance showing that there is a need for broadening the investigations. This study has been carried out to further investigate the etiological aspects of these diseases and collect more data on the natural disease dynamics and the inter-species disease transmission that still remain unclear. Twenty-seven samples have been collected from a larger territory, including the Stelvio National Park but also the mountain valleys of the neighboring provinces of Brescia, Bergamo, and Sondrio (Central Alps) during the period 2008–2018. The limited morbidity rate registered, are probably due to the origin of the cases collected during passive surveillance. A higher prevalence of PPV and PV infected animals can be supported by serological data that showed high antibody prevalence toward another epitheliotropic virus (DPV), indicating that the exposure to theses pathogens is common but the virulence is low with a consequent low rate of disease in Odocoileus populations (9).

Our analyses showed typical parapoxvirus-like particles observed by EM in three red deers found dead showing proliferative lesions covered by crusts, ulcers and erosions on the muco-cutaneous junction of nose and lips. The histologic features were consistent with a parapoxvirus infection in all the cases. Molecular analyses confirmed that the identified virus was the parapoxvirus PVNZ based on the B2L gene and the vVEGF sequences and subsequent phylogenetic analyses. The high level of identity with the same genomic regions of all the available sequences from Italy, Germany and New Zealand further confirms that these parapoxvirus strains are all members of the same species. In contrast the vVEGF gene sequences of all the OV strains responsible of the proliferative lesions of the 11 diseased chamois and five ibexes showed the expected level of genomic variability confirming to be highly hyper-variable as previously reported in OV (46, 47). The low genomic variability of PVNZ might suggest a low selection pressure due to a limited number of susceptible host species, it is in fact known that genomic variants can be a response to increase the fitness of a virus to adapt to immune and antiviral response elicited from different host species (48). The larger host range of OV infecting different domestic and wild animal species and humans may have induced adaptive mutations to allow successful colonization of new animal species (49) contributing to its genomic diversity.

After the first demonstration of PVNZ as the causative agent of the disease in cases of pustular stomatitis in red deer (14), Friederichs et al. (11) isolated PVNZ from tonsils of asymptomatic deer showing that the virus may persist in a small number of healthy looking animals within the population. This data lead us to speculate that, despite the low rate of morbidity, PVNZ may be circulating in persistently infected red deer populations as it was reported in calves (50). Thus, only in a small number of cases, likely in association with other stress factors and/or infectious synergic agents, the PVNZ infections of the mucosal epithelium cause a serious debilitation to the affected animals with reduced ability to feed and death as might have happened to the red deer found dead in our study Persistent infections could allow periodical shedding and clinical manifestations that facilitate the virus survival and spread under favorable conditions (11). Poxvirus-borne life-threatening illnesses, involving the upper alimentary tract occurring in a proportion of affected young and adult deer had been attributed to an immunocompromised state (4). In one of the PVNZ pathological samples (1126C) it was possible to demonstrate also the genome of a papillomavirus and the genetic characterization showed a CePV1v co-infection. CePV1 is the causative agent of red deer fibropapillomatosis and it has not been previously reported as coinfectant agent in a PVNZ induced lesion. The presence of CePV1v is compatible with the ability of PVs to cause latent infection (44). Although the mechanism has not yet been elucidated, following infection they can maintain their genome as episome in the basal layer cells. The lack of detection of mature particles by EM in this sample could support such hypothesis. Our results further confirm the simultaneous presence of different epitheliotropic viruses as already reported in animal and human skin lesions (51–53).

CePV1v was associated with single or multiple fibropapilloma collected from the inner limb of five hunted red deer in agreement with previous reports (14, 33). While there is evidence that Bovine papillomaviruses are not restricted to any anatomical site in cattle (45), fibropapillomas of Red deer associated to CePV1 were always localized in the inner hind leg. This constant localization could be the result of a transmission through arthropod vectors that might play a significant role in the ecology of the virus (28). Indeed, a demonstration of roe deer papillomavirus (CcaPV) DNA in deer keds (*Lipoptena cervi*) and ticks (*Ixodes ricinus*) was reported (29).

The CePV1v confirms to be associated to all the red deer fibropapillomas as previously pointed out and our data further confirm that the pathogen is circulating in Central Alps and in the Stelvio Park being only sporadically reported. The detection of the nucleotide deletion in the L2 gene in all samples is confirmed to be a genomic distinctive characteristic of the CePV1v supporting its classification as a variant of the CcaPV1 that infects roe deer.

More evidence of PPV/PV co-infections is given by the detection of BPV1 and BPV2 in contagious ecthyma samples of two chamois and two ibexes confirming what was previously found in the healthy skin of chamois and ibexes in the Italian Alps (54). The presence of BPV in PPV induced lesions of chamois and ibex poses the question on its pathogenetic role. Poxvirus and Papillomavirus have been simultaneously detected also in skin lesions of cattle (55, 56), birds (57), and humans (51), suggesting that both viruses can complete a replication cycle in the same lesion, providing important data on the host-range of these infectious agents. The skin is the primary barrier to the external environment where groups of viruses sharing cell tropism may be present and not necessarily indicative of a pathology, instead co-infections may represent a degree of commensal interactions (58). Previous report (54) identified bovine papillomavirus DNA in the healthy skin of wild ruminants, documenting coinfection of BPV-1 and CePV1v in healthy red deer in Italy.

Another PPV and PV coinfection was identified in a chamois ecthyma lesion where CePV1v was detected only by molecular biology. This data confirms the ability of this further viral species of the Delta PV genus to infect different animal species beside its natural host. The mechanism of inter species transmission of CePV1v should be studied further in the light of the fact that other Delta papillomaviruses have already shown to be transmissible even to phylogenetically distant animal species thus host tropism of papillomaviruses is not as species-specific as previously thought (22). The genetic characterization of the PPV infecting chamois and ibex suggested that OV has an ecological niche in chamois and ibex as reported for other wildlife species worldwide (39). Nevertheless, phylogenetic analysis of the conserved B2L gene showed that all strains of chamois and ibex grouped with other isolates from sheep and goats instead of clustering by animal species confirming the high genomic variability of the OV species and the susceptibility of wildlife species. In the study area, domestic livestock share pastures with wildlife and it's not uncommon to observe goats among ibexes during the summer season, suggesting the possibility for viral spread among domestic and wild animals.

The same results were obtained by phylogenetic analysis performed on the variable virulence genes vIL10 and GIF that suggested a diverse OV population spreading among chamois and ibex of the Stelvio Park and Central Alps. It was also possible to identify a NZ7 like VEGF variant gene in the isolate from chamois 257, this variant is less widespread and more conserved and often associated with proliferative forms of contagious ecthyma of sheep and goats (40, 59).

*Dermatophilus congolensis* was isolated from one chamois presenting a clinical condition generally referred to as strawberry footrot (43). This animal was in poor general condition presenting massive proliferative OV lesions over the muzzle which might have predisposed the subject to infection and eventually led to the development of severe disease form. Dermatophilosis and contagious ecthyma in wildlife share clinical signs and predisposing factors like traumatic abrasions so they can co-infect host's skin. Due to their zoonotic potential it is important to identify and report these skin diseases (14, 60–63). The persistence of PPV and other zoonotic pathogens in Alpine wild ruminants indicates a source for virus transmission to susceptible livestock and hunters (12, 19, 20, 64). Farmers and veterinarians represent high-risk groups, as their repeated contact with livestock and wild animals expose them to a possible infection. Our findings provide a further example of wildlife animals playing a role as reservoir of zoonotic diseases. Taken all together our results add further information on the microbial populations inhabiting the skin of wild ruminants. Recent studies have demonstrated that interactions between the skin microbiome and the immune system can maintain a healthy skin vs. the establishment of a disease status (65). Metagenomic analyses would be required to further characterize the skin microbiome of these animal species to better understand their cutaneous ecosystem.

## Conclusion

This study reports skin epithelitropic lesions of wild ruminants describing pathologiacal finding and the viral associated agents like CePV1v and PVNZ in red deer and OV in chamois and ibex. Sequences obtained from PPV affected red deer revealed a high degree of identity between individuals examined suggesting that PVNZ is strictly species specific. On the other hand, CePV1v was detected as infectant agent in fibropapilloma of red deer but also as coinfectant of a PPV lesion in a chamois. This evidence leads us to speculate that, like other delta papillomavirus, also CePV1v could gain new hosts and change the epidemiology of an infection that it is only sporadically reported. The high genomic variability of OV affecting chamois and ibex suggests the possibility of transmission of OV from wildlife to livestock. Wild ruminants might be a further source of viruses during the summer season for domestic ruminants and humans. These findings show that active surveillance plan are indeed crucial to collect data on the real spread of epitheliotropic infections in wildlife species and domestic target hosts.

## Data Availability Statement

The original contributions presented in the study are included in the article/supplementary files, further inquiries can be directed to the corresponding author/s.

## Ethics Statement

Ethical review and approval was not required for the animal study because samples were taken from carcasses of necropsied animals, during the diagnostic activities of the IZSLER. In fact, chamois and ibex found dead are commonly submitted to IZSLER and examined in the framework of the regional health monitoring and control plan for wildlife (DDG, 5 December 2012, no. 11358). A certain number of red deer were randomly culled during a program aimed at controlling its population density in the Stelvio National Park (Lombardia Region). Moreover, wild ungulates are legally selective-hunted during the period September-December. In accordance with Italian Law (N. 157 of 11/02/1992), hunters must carry culled wild ruminants to the control centers where age, sex and morpho-biometric measurements are registered and gross lesions inspection of carcass and organs are also performed.

## Author Contributions

LG and FS performed genetic characterization and molecular analyses and wrote the manuscript. GC performed genetic characterization and molecular analyses. AB and IB performed necropsy, coordinated field sampling, and contributed to the preparation of the manuscript. LRG performed histopathological investigation. AL and DL performed electron microscopy and contributed to the preparation of the manuscript. AS assisted in project conception, wrote and edited various parts of the manuscript.

## Conflict of Interest

The authors declare that the research was conducted in the absence of any commercial or financial relationships that could be construed as a potential conflict of interest. The reviewer KE declared a past co-authorship with one of the authors AL, to the handling editor.
